# Isoflurane treatment for refractory and super-refractory status epilepticus in dogs

**DOI:** 10.3389/fvets.2024.1338894

**Published:** 2024-03-08

**Authors:** Eirini Sarpekidou, Georgios Polyzois, Virginia Papageorgiou, Ioannis Savvas, Zoe Polizopoulou, George Kazakos

**Affiliations:** Companion Animal Clinic, School of Veterinary Medicine, Faculty of Health Sciences, Aristotle University of Thessaloniki, Thessaloniki, Greece

**Keywords:** dogs, status epilepticus, refractory, super-refractory, emergency, isoflurane

## Abstract

**Introduction:**

Resistant epileptic episodes, such as refractory status epilepticus (RSE) and super-refractory status epilepticus (SRSE), are neurological emergencies that require immediate medical treatment. Although inhalational anesthetics, such as isoflurane (ISO), have been proposed as a means of seizure control in dogs, there is currently a lack of both experimental and clinical studies on this subject.

**Study design:**

This is a retrospective clinical study.

**Methods:**

Records of dogs that received ISO for the management of RSE and SRSE during their intensive care unit (ICU) hospitalization at the Companion Animal Clinic of the Aristotle University of Thessaloniki were included in the present study. The study period spanned from February 2013 to March 2023. Dogs were identified as responders (R) when RSE/SRSE ceased after ISO administration, and the dogs were successfully discharged from the ICU after ISO discontinuation. Dogs were identified as non-responders (NR) when RSE/SRSE ceased after ISO administration, but RSE/SRSE reoccurred after ISO discontinuation. Additional data about the number and time of ISO cycles, the time of ICU hospitalization, the side effects of ISO administration, and an additional administration of antiepileptic drugs (AEDs) and anesthetic drugs were also recorded.

**Results:**

A total of 20 dogs with 26 recorded RSE/SRSE episodes and 26 anesthetic cycles with ISO were included in the present study. The clinical termination of seizure activity was achieved 100% (26/26) in all episodes. In 73.1% (19/26) of the episodes, ISO administration resulted in successful RSE/SRSE treatment. Poor outcome was recorded in 26.9% (7/26) of the episodes because RSE/SRSE reoccurred after ISO discontinuation, and the dogs were euthanatized or died due to cardiac arrest. Inspiratory ISO ranged between 0.5 and 4.0%. The median time of the anesthetic cycles with ISO was 12.67 h (4.00–62.00). The median duration of the ICU hospitalization was 48.00 h (24.00–120.00). At least one ISO-related side effect was recorded in 23 out of 26 (88.5%) episodes.

**Conclusion:**

To the authors’ knowledge, this is the first clinical study that addresses the administration of ISO for RSE/SRSE treatment in dogs. The use of ISO may be beneficial in terminating RSE/SRSE; however, further prospective studies are necessary to confirm this observation.

## Introduction

Status epilepticus (SE) is characterized by continuous epileptic seizures for over 5 min or multiple episodes (two or more) of epileptic seizures without consciousness reacquisition among them (1–3). Resistant epileptic episodes are defined as refractory status epilepticus (RSE) and super-refractory status epilepticus (SRSE). RSE is the state of epileptic activity that continues for more than 30 min and cannot be terminated with first- and second-line antiepileptic drugs (AEDs). Anesthetic drugs are administered when the epileptic activity reoccurs or continues after the first- and second-line of treatment. If SE continues for more than 24 h, then it is designated as SRSE (4, 5).

In human medicine, intravenous anesthetic drugs, such as ketamine (KET) or propofol (PPF), are administered for the management of refractory epileptic episodes. Therapeutic coma is also suggested in human medicine, in patients with drug-resistant epileptic activity (6–9). Inhalational anesthesia with ISO is recommended in human RSE and SRSE cases (10–17). The mechanism of action of ISO remains only partially understood, but it may be related to ion channels by binding to protein sites and by altering the function of nicotinic acetylcholine, GABAA (γ-aminobutyric acid type A) and glutamate receptors, which belong to the fast synaptic neurotransmitter receptors (18, 19). There are still scarce studies on humans for the use of ISO on epileptic activity termination in refractory states (10–17).

According to veterinary literature, most epileptic dogs will need emergency care in order to terminate SE at least once in their life as 60% of them will suffer one status epilepticus episode (2). Resistant epileptic episodes remain a challenging condition and general anesthetics may be required in order to control the refractory seizure activity (2–5). In veterinary medicine, there are no clinical published data about the use of ISO in epileptic animals. There are only two experimental studies in cats; in these studies, experimentally induced seizure activity was controlled by inhalational anesthetic administration (20, 21). Inhalational anesthesia is recommended by many authors in veterinary textbooks in RSE or SRSE, when AEDs, non-anesthetic drugs, and injectable anesthetic drugs fail, but there is a lack of clinical trials and recorded cases in dogs (2–5). Although ISO administration is recommended in the latest consensus statement on the management of cluster seizures and status epilepticus, no specific guidelines and instructions about the use of ISO in refractory epileptic episodes are mentioned, due to the lack of veterinary clinical published data (5).

This study aimed to evaluate the outcomes of seizure termination in dogs with refractory seizure activity after inhalational anesthesia with ISO and to identify any side effects.

## Materials and methods

In the present retrospective study, ICU records of dogs admitted to the Companion Animal Clinic of the Aristotle University of Thessaloniki between February 2013 and March 2023 were reviewed. Data on age, sex, weight, and breed were collected from the ICU records (Table 1).

**Table 1:** Demographic and disease characteristics of the dogs.

Dogs (Patient No.)	Age (Years)	Sex	Weight (Kg)	Breed	Epilepsy type	Diagnosis	AEDs	SRSE/RSE episodes	Existing epilepsy
1	1.5	Male	8	Cavalier King Charles Spaniel	StE	Hydrocephalus	Yes	2	Yes
2	4	Male	6.5	Maltese Cross	CE	Cryptogenic	No	1	No
3	10	Male	20	French Bulldog Cross	StE	Neoplasia	No	2	No
4	5	Male neutered	2.5	Yorkshire Terrier	StE	MUO	Yes	2	Yes
5	2.5	Female neutered	42.5	Cane Corso	StE	Toxoplasmosis	No	1	No
6	1.5	Female	3.5	Yorkshire Terrier	RE	Portosystemic shunt	No	1	No
7	3	Female neutered	3	Yorkshire Terrier	StE	MUO	Yes	1	Yes
8	8	Male neutered	31	Mongrel	CE	Cryptogenic	No	1	No
9	10	Male	17	Mongrel	StE	Frontal lobe neoplasia-postoperative	Yes	1	Yes
10	4	Male	51	Dog de Bordeaux	CE	Cryptogenic	No	1	No
11	7.5	Male	23	GSD cross	StE	Meningioma Postoperative	No	1	No
12	5	Male	2	Yorkshire Terrier	StE	MUO	No	1	No
13	1.5	Male	33	Pit Bull	CE	Cryptogenic	Yes	1	Yes
14	7	Female	24	GSD cross	CE	Cryptogenic	No	1	No
15	8	Male	32	Mongrel	CE	Cryptogenic	No	1	No
16	4	Male	10	Jack Russell	IE	Idiopathic epilepsy	Yes	4	Yes
17	3.8	Male neutered	3.8	Yorkshire Terrier	ΙΕ	Idiopathic epilepsy	No	1	No
18	5	Male	4.2	Yorkshire Terrier	RE	Portosystemic shunt	No	1	No
19	7	Male	23.2	Beagle	StE	Intra-arachnoid cyst + necrotic lesion	Yes	1	Yes
20	4	Male	32	Mongrel	IE	Idiopathic epilepsy	Yes	1	Yes
*n* = 20	Mean age 5.1 (Standard deviation 2.66)		Median weight 18.5 (min 2.0–max 51.0)					*n* = 26	

Additional information about the underlying cause of the epileptic episode was retained from the clinical records and according to the diagnostics that were conducted before or after the RSE/SRSE episode. The dogs were categorized into the following four different groups according to the possible underlying diagnosis. Structural epilepsy (StE) was diagnosed in case any tumor, malformation, or lesion of the brain was reported, idiopathic epilepsy (IE) was diagnosed in case no pathological underlying cause could be determined, reactive epilepsy (RE) was diagnosed in cases of a portosystemic shunt, and cryptogenic epilepsy (CE) was diagnosed in dogs when the owners refused further diagnostic evaluation leading to unidentified underlying pathology.

Cases that met the following criteria were included: dogs with RSE or SRSE unresponsive to AEDs and intravenous anesthetic drugs treated with ISO administration. In order to be included in the study, dogs should have received a combination of AEDs including the dogs for whom PPF administration—boluses or CRI—was initiated without successful RSE/SRSE treatment, e.g., seizure reoccurred after PPF discontinuation (Table 2). All patients included should be intubated after PPF administration and then receive ISO in an oxygen and air mixture.

**Table 2:** AEDs before ISO administration.

Patient no.	RSE/SRSE Episode *n* = 26	Long-term AEDs	AEDs during hospitalization and the line of administration before ISO
1	1	PB 2.5 mg/kg BID	1st MDZ CRI 0.3–0.4 mg/kg/h 2nd PB 2.5 mg/kg BID 3rd PPF CRI 0.3–0.4 mg/kg/min
	2	PB 2.5 mg/kg BID	1st MDZ CRI 0.3–0.4 mg/kg/h 2nd PB 2.5 mg/kg BID 3rd PPF CRI 0.3–0.4 mg/kg/min
2	3	–	1st MDZ CRI 0.2–0.4 mg/kg/h 2nd PB 4 mg/kg QID 3rd PPF boluses 4 mg/kg
3	4	_–_	1st MDZ CRI 0.2–0.4 mg/kg/h 2nd PB 4 mg/kg QID 3rd PPF boluses 4 mg/kg and CRI 0.3–0.5 mg/kg/min
	5		1st MDZ CRI 0.2–0.4 mg/kg/h 2nd PB 4 mg/kg QID 3rd PPF boluses 4 mg/kg and CRI 0.3–0.5 mg/kg/min
4	6	PB 2 mg/kg BID	1st MDZ CRI 0.4–0.5 mg/kg/h 2nd PB 2.5 mg/kg BID, LEV 60 mg/kg SID, and 25 mg/kg TID 3rd PPF boluses 4 mg/kg
	7	PB 2 mg/kg BID	1st MDZ CRI 0.4–0.5 mg/kg/h 2nd PB 2.5 mg/kg BID, LEV 60 mg/kg SID, and 25 mg/kg TID 3rd PPF boluses 4 mg/kg
5	8	–	1st MDZ CRI 0.3–0.4 mg/kg/h 2nd LEV 60 mg/kg SID and 20 mg/kg TID 3rd PPF CRI 0.3–0.5 mg/kg/min
6	9	–	1st LEV 60 mg/kg SID and 20 mg/kg TID 2nd PPF CRI 0.3–0.4 mg/kg/min
7	10	LEV 20 mg/kg TID	1st MDZ CRI 0.2–0.4 mg/kg/h 2nd PB 4 mg/kg QID, levetiracetam 25 mg/kg TID 3rd PPF CRI 0.3–0.5 mg/kg/min
8	11	–	1st MDZ CRI 0.2–0.4 mg/kg/h 2nd LEV 60 mg/kg SID and 20 mg/kg TID 3rd KBr 100 mg/kg QID 4th PPF boluses 4–6 mg/kg
9	12	PB 2.5 mg/kg BID	1st MDZ CRI 0.4–0.5 mg/kg/h 2nd PB 2.5 mg/kg BID 3rd PPF CRI 0.3–0.5 mg/kg/min
10	13	–	1st MDZ CRI 0.4 mg/kg/h 2nd PB 4 mg/kg QID 3rd PPF boluses 5 mg/kg
11	14	–	1st MDZ CRI 0.2–0.4 mg/kg/h 2nd PB 4 mg/kg QID 3rd PPF CRI 0.3–0.5 mg/kg/min
12	15	–	1st MDZ CRI 0.3 mg/kg/h 2nd PB 4 mg/kg QID 3rd PPF boluses 4 mg/kg
13	16	Levetiracetam 20 mg/kg TID	1st DZP rectal administration 2nd MDZ CRI 0.3 mg/kg/h 3rd PB 4 mg/kg QID 4th PPF CRI 0.5–0.6 mg/kg/min
14	17	–	1st MDZ CRI 0.2–0.4 mg/kg/h 2nd PB 4 mg/kg QID 3rd PPF boluses 4 mg/kg and CRI 0.3 mg/kg/min
15	18	–	1st MDZ CRI 0.2–0.4 mg/kg/h 2nd PB 4 mg/kg QID 3rd PPF CRI 0.4–0.5 mg/kg/min
16	19	–	1st MDZ CRI 0.3–0.4 mg/kg/h 2nd PB 4 mg/kg QID 3rd PPF bolus 4 mg/kg
	20		1st MDZ CRI 0.3–0.4 mg/kg/h 2nd PB 4 mg/kg QID 3rd PPF bolus 4 mg/kg
	21	PB 2 mg/kg BID	1st PB 2.5 mg/kg BID 2nd KBr 100 mg/kg QID 3rd PPF CRI 0.3 mg/kg/min
	22	PB 2.5 mg/kg BID KBr 40 mg/kg SID	1st PB 2.5 mg/kg BID 2nd MDZ CRI 0.4–0.5 mg/kg/h, LEV 60 mg/kg SID, and 25 mg/kg TID 3rd KBr 40 mg/kg SID 4th PHT10mg/kg 5th PPF CRI 0.4–0.6 mg/kg/min
17	23	–	1st MDZ CRI 0.3–0.4 mg/kg/h 2nd PB 4 mg/kg QID, LEV 60 mg/kg SID, and 25 mg/kg TID 3rd PPF CRI 0.4–0.5 mg/kg/min
18	24	–	1st LEV 60 mg/kg SID and 20 mg/kg TID 2nd PPF CRI 0.3–0.4 mg/kg/min
19	25	PB 2.5 mg/kg, LEV 25 mg/kg TID, IMP 30 mg/kg BID	1st LEV 25 mg/kg TID 2nd MDZ CRI 0.4–0.5 mg/kg/h 3rd PHT 10 mg/kg 4th IMP 30 mg/kg BID 5fth PPF 0.3–0.5 mg/kg/min
20	26	PB 2.5 mg/kg BID LEV 25 mg/kg TID	1st PB 2.5 mg/kg BID and LEV 25 mg/kg TID 2nd KBr 100 mg/kg QID 3rd PPF 0.5 mg/kg/min 4th KET bolus 1 mg/kg then CRI 1 mg/kg/h and DEX bolus 3 mg/kg then CRI 3 μg/kg/h

Inspiratory ISO percentage was documented according to the ICU records, and the minimum and maximum values were recorded (Table 3). Additionally, any side effects associated with the anesthesia induction and maintenance were recorded. The minimum and maximum values of hypothermia and hypercapnia were documented. The presence of hypotension accompanied by the drugs administered was accessed. Possible respiratory infections were also evaluated (Table 4).

**Table 3:** RSE/SRSE treatment with ISO and outcomes.

Patient no	Anesthetic cycle	Long-term AEDs	AEDs during hospitalization	Cycles of anesthesia with ISO during hospitalization	ISO %	Anesthesia cycle duration (h)	Time without recurrence in the ICU after ISO (h)	Duration of ICU hospitalization	RSE/SRSE episode termination YES/NO	Final outcome
1	1	PB	PB MDZ PPF	1st	1.0–1.5	4.0	8.0	48.0	Yes	–
	2	PB	PB MDZ PPF	2nd	1.0–2.5	8.0	0		No	NR—cardiac arrest
2	3	–	PB MDZ PPF	1st	0.5–1.0	15.0	0	48.0	No	NR—cardiac arrest
3	4	–	PB MDZ PPF	1st	1.0	18.0	4.0	96.0	Yes	–
	5	–	PB MDZ PPF	2nd	1.5–2.0	48.0	0		No	NR—euthanasia
4	6	PB	PB LEV MDZ PPF	1st	1.0–2.0	62.0	0.5	96.0	Yes	–
	7	PB	PB LEV MDZ PPF	2nd	1.2–2	6.0	0		No	NR—euthanasia
5	8	–	LEV MDZ PPF	1st	2.0	4.4	0	24.0	No	NR—euthanasia
6	9	–	LEV PPF	1st	0.8–1.5	23.0	0	48.0	No	NR—cardiac arrest
7	10	LEV	PB LEVMDZ PPF	1st	1.2–3.0	20.0	0	48.0	No	NR—euthanasia
8	11	–	LEV MDZ PPF KBr	1st	1.5–2.0	10.5	34.0	48.0	Yes	R
9	12	PB	PB MDZ PPF	1st	2.0–2.6	4.0	27.0	48.0	Yes	R
10	13	–	PB MDZ PPF	1st	1.2–2.0	5.0	32.0	72.0	Yes	R
11	14	–	PB MDZ PPF	1st	1.7–2.3	9.0	42.0	96.0	Yes	R
12	15	–	PB MDZ PPF	1st	1.5–2.0	7.0	30.0	72.0	Yes	R
13	16	LEV	PB DZP MDZ PPF	1st	1.0	4.0	48.0	96.0	Yes	R
14	17	–	PB MDZ PPF	1st	1.5–2.0	8.0	36.0	72.0	Yes	R
15	18	–	PB MDZ PPF	1st	0.8–2.1	13.0	35.0	48.0	Yes	R
16	19	–	PB MDZ PPF	1st	1.2–2.0	8.5	5.0	120.0	Yes	–
	20	–	PB MDZ PPF	2nd	1.0–2.5	18.0	34.0		Yes	R
	21	PB	PB KBr PPF	1st	1.5	5.0	32.0	48.0	Yes	R
	22	PB, KBr	PB MDZ KBr LRVPHT PPF	1st	1.5–2.3	13.0	48.0	72.0	Yes	R
17	23	–	PB LEV MDZ PPF	1st	1.0–1.5	4.0	26.0	48.0	Yes	R
18	24	–	LEV PPF	1st	0.8–1.0	4.0	36.0	48.0	Yes	R
19	25	PB LEV IMP	LEV MDZ PHT IMP PPF	1st	1.5	4.0	40.0	48.0	Yes	R
20	26	PB, LEV	PB LEV KET DEX KBr PPF	1st	2.0–4.0	4.0	48.0	72.0	Yes	R
	*n* = 26					12.67 h (min 4.00–max 62.00)	28.50 h (min 0.0–max 48.0)	48.00 (min 24.00–max 120.00)		

**Table 4:** Side effects of ISO administration.

Patient	Anesthesia cycle	RSE-SRSE episode No.	Cycles of anesthesia during hospitalization	Isoflurane % inspiratory	Total anesthesia time (h)	Hypothermia (°C)	Hypotension	Respiratory infection	PCO_2_/EtCO_2_ mmHg	RSE/SRSE episode termination	Outcome
1	1	1	1st	1.0–1.5	4.0	37.2	Yes	No	NA	Yes	NR—euthanasia
	2	2	2nd	1.0–2.5	8.0	37.4	Yes	No	NA	No	
2	3	3	1st	0.5–1.0	15.0	36.3	No	No	PCO_2_ min 27.4–max 71.9	Yes	NR—cardiac arrest
3	4	4	1st	1.0	18.0	35.0	No	Yes	PCO_2_ min 54.6–max 67.3	Yes	NR—euthanasia
	5	5	2nd	1.5–2.0	48.0	36.4	No	Yes	PCO_2_ min 58–max 73.1	No	
4	6	6	1st	1.0–2.0	62.0	34.0	Yes	No	EtCO_2_ min 41–max 45	No	NR—euthanasia
	7	7	2nd	1.2–2	6.0	35.2	No	No	EtCO_2_ min 43–max 45	No	
5	8	8	1st	2.0	4.40	37.6	Yes	No	EtCO_2_ min 42–max 48	Yes	NR—euthanasia
6	9	9	1st	0.8–1.5	23.0	37.5	Yes	No	EtCO_2_ min 35–max 45	No	NR—cardiac arrest
7	10	10	1st	1.2–3.0	20.0	37.6	Yes	No	EtCO_2_ min 35–max 55	No	NR—euthanasia
8	11	11	1st	1.5–2.0	10.5	No	No	No	NA	Yes	R
9	12	12	1st	2–2.6	4.0	No	No	No	NA	Yes	R
10	13	13	1st	1.2–2.0	5.0	37.3	No	No	NA	Yes	R
11	14	14	1st	1.7–2.3	9.0	36.0	No	No	EtCO_2_ min 33.8–max 34.5	Yes	R
12	15	15	1st	1.5–2	7.0	33.1	No	No	NA	Yes	R
13	16	16	1st	1.0	4.0	37.0	No	No	PCO_2_ min 45.7–max 51.6	Yes	R
14	17	17	1st	1.5–2.0	8.0	36.9	No	No	EtCO_2_ min 45–max 48	Yes	R
15	18	18	1st	0.8–2.1	13.0	37.9	No	No	EtCO_2_ min 36–max 44.8	Yes	R
16	19	19	1st	1.2–2.0	8.5	35.2	No	No	EtCO_2_ min 33–max 60	Yes	R
	20	20	2nd	1.0–2.5	18.0	35.7	No	No	EtCO_2_ min 38–max 57	Yes	
	21	21	1st	1.5	5.0	35.4	No	No	NA	Yes	R
	22	22	1st	1.5–2.3	13.0	35.8	No	No	EtCO_2_ min 58–max 64	Yes	R
17	23	23	1st	1.0–1.5	4.0	36.5	Yes	No	NA	Yes	R
18	24	24	1st	0.8–1.0	4.0	36.5	No	No	EtCO_2_ min 31–max 57	Yes	R
19	25	25	1st	1.5	4.0	No	No	No	EtCO_2_ min 39–max 53	Yes	R
20	26	26	1st	2.0–4.0	4.0	37.4	No	No	EtCO_2_ min 55–max 60	Yes	R
				0.5–4.0%	12.67 (min 4.00–max 62.00)	23/26–88.5%	7/26–26.9%	2/26–7.7%	PCO_2_ min 27.4–max 73.1 mmHg EtCO_2_ min 31–max 64 mmHg		

Dogs as individual cases (7/20) and as medical records/hospitalization (7/22, since dog 16 was hospitalized three times) were defined as non-responders (NR) in cases when refractory epileptic activity reoccurred after ISO discontinuation and anesthetic recovery attempt. Subsequently, NR dogs could not be discharged and were euthanized or died during RSE/SRSE management. Dogs as individual cases (13/20) and as medical records/hospitalization (15/22) were characterized as responders (R) when RSE/SRSE was terminated after ISO discontinuation and anesthetic recovery attempt, and the patient was neurologically intact and could be discharged from the ICU. Seizure termination could only be decided clinically, in the absence of seizure activity and mentation restoration.

Patients with incomplete records referring to outcome and AED administration before or during the admission were excluded.

Data on age, weight, and hours of ISO administration were evaluated for normality, and descriptive statistics were produced.

## Results

### Study population

In total, 22 ICU records in 20 dogs were included in the present retrospective study. The animals were Cavalier King Charles Spaniel (1), Maltese Cross (1), French Bulldog Cross (1), Yorkshire Terrier (6), Cane Corso (1), Dogue de Bordeaux (1), German Shepherd Dog cross (2), Pit Bull (1), Jack Russell (1), Beagle (1), and mongrel (4) dogs. Thirteen were intact male dogs, three were male neutered dogs, two were intact female dogs, and two were female neutered dogs. The dogs had a mean (range) age of 5.1 (SD 2.66) years and a median weight of 18.5 (2.0–51.0) kg. Yorkshire Terrier dogs were the most represented, followed by mongrel and German Shepherd cross dogs. Demographic and disease characteristics of the dogs are summarized in Table 1. The underlying cause was defined as structural epilepsy (StE) in 9 of 20 cases (45%), cryptogenic epilepsy (CE) in 6 of 20 cases (30%), idiopathic epilepsy (IE) in 3 of 20 cases (15%), and reactive epilepsy (RE) in 2 of 20 cases (10%). All R dogs included had a single anesthesia record except for one dog (No. 16, Table 1), which was admitted to the ICU with RSE/SRSE three times in a period of 4 months (May–September 2017). The rest of the R dogs were RSE/SRSE-free for 4 months after the episode was terminated. In our study, 8 of 20 dogs (40%) had already been diagnosed with epilepsy and had received one or more AEDs [phenobarbital (PB), levetiracetam (LEV), potassium bromide (KBr), imepitoin (IMP)] and 12 of 20 dogs (60%) were admitted in the ICU with acute epileptic activity without a known history of epilepsy.

### Long-term AEDs and epileptic management before ISO

Before admission, 9 of 22 (41%) dogs received maintenance AEDs. PB was administered in 4 of 9 dogs (44.4%); LEV in 2 of 9 dogs (22.2%); PB and KBr in 1 of 9 dogs (11.1%); PB, LEV, and IMP in 1 of 9 dogs (11.1%); and PB and LEV in 1 of 9 dogs (11.1%). Epileptic management before ISO administration included a combination of AEDs, seizure management, and CRI of anesthetic drugs. The following drugs were included: PB, LEV, IMP, KBr, midazolam (MDZ), diazepam (DZP), phenytoin (PHT), PPF, dexmedetomidine (DEX), and KET (Table 2). PPF boluses and CRI of PPF in doses ranging from 0.3 to 0.6 mg/kg/min were administered for seizure control. Patients who received PPF CRI were intubated, and anesthesia recovery was initiated. In those dogs, epileptic activity reoccurred after PPF discontinuation and thus followed by ISO administration.

### Treatment with ISO

All patients were intubated after PPF boluses of 0.5 mg/kg IV were administered every 30 s followed by additional doses, to effect, i.e., until intubation could be performed. During the beginning of the administration of ISO following induction/intubation, no side effects were observed. Dogs received Lactated Ringers or NS 0.9% solution, and electrocardiography, respiratory rate, SpO2, rectal temperature, and oscillometric blood pressure (NIBP) measurements were continuously monitored and recorded. Additionally, in most of the episodes, end-tidal carbon dioxide (EtCO2) or partial pressure of carbon dioxide (PaCO2) was also measured. In the episodes with hypotension during anesthesia, vasopressors were administered.

### Seizure treatment during ISO administration and the recovery of ISO anesthesia

The clinical termination of seizure activity was achieved 100% (26/26) in all RSE/SRSE episodes, and dogs remained clinically seizure free while anesthetized on ISO. All dogs received one cycle of ISO except for patients 1, 3, 4, and 16 who received a second cycle. Patients 1, 3, and 4 were included in the NR group because the refractory seizure activity reoccurred after ISO discontinuation of 8, 4, and 0.5 h, respectively, after anesthesia recovery. Thus, a second cycle of anesthesia with ISO was initiated, but the dogs were euthanized or died from cardiac arrest during the RSE/SRSE management. Patient No. 16 responded to the initial ISO administration and recovered from the anesthetic cycle and was seizure free for 5 h, but refractory seizure activity reoccurred and the dog was again anesthetized with ISO. After the second anesthesia cycle, the dog recovered without seizure activity and was discharged from the ICU; thus, patient No. 16 was included in the R group. In total, 26 cycles of ISO administration in 22 hospitalizations in 20 patients were recorded. In 19 of 26 (73.1%) cycles of ISO administration, a seizure-free recovery from anesthesia could be achieved. In 7 of 26 (26.9%) cycles, the seizure activity reoccurred every time ISO was discontinued (the first cycle in patients 1, 3, and 4 and the second anesthetic cycle in patients 2, 5, 6, and 7). These patients were euthanized or died from cardiac arrest.

RSΕ/SRSΕ were terminated in 15 of 22 (68%) hospitalizations, and all of the R patients were status free until discharge. In 7 of 22 (32%) hospitalizations, an extubation and recovery attempt was initiated at least once (or multiple times), but the epileptic activity reoccurred. Cardiac arrest was the cause of death in three of seven (43%) NR dogs during RSE/SRSE management, and four of seven NR (57%) dogs were euthanized due to poor prognosis. Favorable outcomes and ICU discharge after RSE/SRSE treatment were reported in 13 of 20 (65%) dogs and poor outcomes in 7 of 20 dogs (35%).

Depending on the available records, inspired ISO concentration ranged from 0.5 to 4 vol%, and inspired oxygen fraction ranged between 50 and 100% and was adjusted depending on the patient’s requirements. In most hospitalizations, in 18 of 22 (82%) patients, one cycle of anesthesia was applied, of which 4 of 18 (22.2%) were NR and 14 of 18 (77.7%) were R. In 4 of 22 (18%) hospitalizations, two anesthesia cycles were applied, with three of four (75%) being NR and one of four (25%) being R. The median time of the anesthetic cycles was 12.67 (4.00–62.00) h. The median time without recurrence in the ICU after inhalational anesthesia recovery was 28.50 (min 0.0–max 48.0) h. The median time of ICU hospitalization is 48.00 (24.00–120.00) h. ISO (cycle) was administered for at least 4 h before discontinuation. Then, ISO was gradually reduced. If epileptic activity reoccurred after the ISO reduction, ISO concentration was increased. If the dog remained seizure free, the anesthetic recovery was performed. In dogs that required more than 4 h of ISO administration, it was continued for at least 1 h, before the ISO was reduced again.

### Side effects associated with isoflurane administration

The side effects of isoflurane are summarized in Table 4. In 23 of 26 (88.5%) episodes, at least one ISO-related side effect was recorded. In 23 of 26 (88.5%) episodes, hypothermia occurred during the anesthetic cycle with ISO, and the mean (range) temperature was 36.3°C (33.1–37.9). In 7 of 26 (26.9%) episodes, hypotension occurred during anesthesia. Six of seven (85.7%) were NR, and one of seven (14.3%) were R and received vasopressors. In (No. 3) 1 of 20 (5%) patients, aspiration pneumonia was reported. Additionally, in 18 of 26 anesthesia records, PCO_2_ or EtCO_2_ was recorded during anesthesia; in the rest of the records, these values were designated as missing. In most of the episodes, hypercapnia in non-mechanical ventilated patients was observed. Mechanical ventilation was initiated in episodes with PCO_2_ or EtCO_2_ values above 55 mmHg elevation for more than 10 min. In NR patients, the PCO_2_ ranged from 27.4 to 73.1 mmHg and EtCO_2_ from 35 to 55 mmHg. In R patients, EtCO_2_ ranged from 31 to 64 mmHg and the PCO_2_ from 45.7 to 51.6 mmHg (patient No. 13).

## Discussion

Emergency medical treatment should be considered in the case of continuous epileptic activity because it may lead to a refractory state (3–5). Delayed intervention can cause life-threatening conditions due to an increase in intracranial pressure, cerebral blood flow disturbances, disruption of the cerebral protective mechanism, brain tissue necrosis, and systematic blood circulation disorders, such as arterial hypertension (3). These complications increase the morbidity and mortality rates of epileptic humans and animals; therefore, seizure termination is of utmost importance (2, 3, 10).

Injectable or inhalational general anesthetics are suggested for RSE/SRSE management due to the anticonvulsant and neuroprotective properties of some general anesthetics. General anesthetics, such as PPF and KET, in boluses or CRI, are commonly administered as third-line drugs, in RSE/SRSE. Ketamine as an N-methyl-D-aspartic acid (NMDA) antagonist may reduce neuronal excitability and contribute to refractory epileptic activity suppression and may have neuroprotective properties (4, 22, 23). The anticonvulsant properties of PPF are based on blocking NMDA receptors and alterations in GABA receptors leading to seizure cessation (8).

ISO administration is recommended in dogs with RSE/SRSE, but there are no clinical studies published to date (4, 5). Our study is based on evidence taken from the human literature about the anticonvulsant properties of ISO. The mechanism of action of ISO is not completely clear. The main effect of action may be on the fast synaptic neurotransmitter receptors such as nicotinic acetylcholine, GABAA, and glutamate receptors. Additionally, by binding to protein sites, ISO enhances the inhibition of neurotransmitter-controlled ion channels (18, 19).

This is the first clinical retrospective study in veterinary medicine to evaluate isoflurane administration and RSE/SRSE treatment in dogs. In all recorded episodes, refractory epileptic activity was terminated by ISO administration after PPF doses for intubation. In more than half of the dogs included in the present study, the underlying cause of epileptic activity was associated with brain lesions—StE (hydrocephalus, brain neoplasia, MUO, intra-arachnoid cyst, and necrotic lesion) and metabolic disorders—RE (portosystemic shunt). The underlying pathology could explain the resistant form of the RSE/SRSE episodes and the transition to a refractory epileptic state after the initial seizure termination with first- and second-line AEDs or anesthetic drugs. Although the fact that one of three of the dogs had an unidentified etiology for seizure activity—CE—, we could suspect metabolic and brain tissue disorders by the resistant epileptic form of the episodes. Only two dogs had RSE/SRSE associated with IE which may represent the resistant form of IE unresponsive to the common seizure activity management. Patient No. 16 suffering from IE was admitted to the ICU 3 times in 4 months, RSE/SRSE was successfully terminated after ISO administration, and the dog was discharged. Seizure activity reoccurred in 7 of 22 dogs (32% NR) during ICU hospitalization. In those dogs, RSE/SRSE was stopped when ISO administration was initiated, and the dogs were seizure free during anesthesia. Patients 2, 5, 6, and 7 could not be discontinued from ISO because, in every anesthetic recovery attempt, seizure activity reoccurred. Patients 1, 3, and 4 were recovered from the first anesthetic cycle with ISO and were seizure free, but several hours after ISO discontinuation, refractory epileptic activity reoccurred, and the dogs were anesthetized again with ISO, aiming RSE control. The recurrence of refractory epileptic activity in the aforementioned cases could be attributed to the fact that brain tissue lesions were reported as the underlying etiology. All NR dogs died from cardiac arrest or were euthanized. In human medical literature, ISO administration is suggested in RSE, but only a few published single case or case series reports were available until recent times, resulting in controversial results (11–17, 24–29). In a systematic review conducted by Zeiler et al., the percentage of SE termination after inhalational anesthetic administration was 92.9% in adults and 94.4% in pediatric patients, but in some cases, epileptic activity reoccurred. (30). In a more recent multicenter retrospective study about SE treatment, in patients with RSE/SRSE, after ISO administration the success rate was 51% based on electroencephalography (EEG) or ICU discharge (10). Moreover, in an experimental study conducted on anesthetized cats, experimentally induced epileptic activity monitored by EEG was successfully terminated with inhalational anesthesia with ISO and sevoflurane (20, 21).

SE medical management is recommended and includes AEDs and non-anesthetic and anesthetic drugs (2–5). In dogs with RSE/SRSE, conventional treatment options are limited to intravenous, rectal, oral, and intranasal drug administration (4, 5). Intravenous KET is suggested if conventional management of refractory epileptic activity with AEDs and benzodiazepine is ineffective (4, 5). The administration of intravenous DEX and KET in three dogs presented with SRSE led to successful epileptic activity termination (22). In a retrospective study including SE, RSE, and cluster seizure cases, the epileptic activity was successfully terminated after KET boluses in 12 cases with RSE (23). In the present study, AEDs were administered in all patients accompanied by anesthetic drugs before ISO administration. Ketamine boluses were not administered in the present study except for patient No. 22 who received DEX and KET boluses upon admission (22). ISO was then administered and led to successful refractory seizure control. It is still unknown whether the combination of inhalational and intravenous drugs could lead to better outcomes.

In our study, there are large variations in inspiratory ISO concentration and in the duration of the anesthetic cycle. These variations depended on the decision of the clinician in charge and on different management options available over the period of 10 years. There is also a variability concerning the duration and concentration of ISO administration in human medicine. For refractory epileptic activity, anesthesia duration from 1 h to 104 days is reported (10, 28). Significant variations are also reported in the administered ISO percentage and range from 0.5 to 5% (7, 11–13, 15, 16, 24–29).

ISO administration in RSE/SRSE patients in human medicine has been associated with side effects such as hypothermia, hypotension, respiratory infection, atelectasis, paralytic ileus, deep vein thrombosis, and brain tissue changes such as hippocampal changes and multifocal T2 hyperintensity (10, 15, 30). Hypothermia, hypotension, respiratory infection, and hypercapnia were reported in our study. Hypothermia was the most frequently recorded side effect. In experimental models, severe hypothermia was associated with anticonvulsant properties, but in clinical trials, the results remain controversial (31–35). In human medicine, the long-term administration of ISO can lead to brain changes (12, 15, 17). In our study, we cannot identify these changes because of the lack of postanesthetic evaluation of the brain. Hypotension was developed in NR patients more frequently, and this finding could be attributed to the longer anesthetic ISO cycles in these dogs. Dopamine administration in CRI led to blood pressure restoration without additional actions (36). Dogs under anesthesia may develop hypercapnia (PaCO_2_ > 45 mmHg), although values between 35 and 50 mmHg may be acceptable in anesthetized patients (36). Only few patients that were included in the study developed severe hypercapnia and were mechanically ventilated. No complications of ISO administration leading to the discontinuation of the anesthetic cycle were recorded.

The first limitation of the present study is its retrospective nature. Furthermore, SE treatment was assessed only clinically because of the absence of EEG. Ketamine boluses were not administered in the present study; ketamine administration could possibly affect the outcome of RSE/SRSE termination. Another limitation is the absence of a standardized baseline treatment protocol for seizure activity, thus resulting in a large variation in the duration of ISO administration in our study. Seizure termination was evaluated based on clinical examination and the absence of epileptic activity during ISO administration. RSE/SRSE treatment could be evaluated after anesthesia recovery. No follow-up diagnostic imaging was conducted after the anesthetic cycles with ISO in any of the cases included in the present study. It is unknown whether there was any postanesthetic brain abnormality in these dogs. Moreover, due to financial reasons and acute development of RSE/SRSE, etiological diagnosis is obscure. In some cases, the owners refused to undergo further diagnostic tests, as the outcome was positive. This treatment requires qualified staff and it is expensive, and thus, it can be offered only in veterinary hospital conditions.

To our knowledge, this is the first clinical study in veterinary medicine of the use of ISO with the intent to treat RSE/SRSE in dogs. According to the results of this study, ISO may contribute to seizure termination in cases of refractory epileptic episodes in dogs suffering from RSE/SRSE. Although there is no strong evidence about ISO administration in RSE/SRSE, it seems that it could be helpful in the management of refractory seizure control in dogs. ISO should be further evaluated as a potential treatment of RSE/SRSE. Future researchers taking into consideration the aforementioned limitations may conduct a prospective study that could strengthen the evidence on the effect of ISO administration on RSE/SRSE treatment in dogs.

## Data availability statement

The raw data supporting the conclusions of this article will be made available by the authors, without undue reservation.

## Ethics statement

Ethical approval was not required for the studies involving animals in accordance with the local legislation and institutional requirements because to our institution, there is no need for ethical approval in conducting a retrospective study. Written informed consent was obtained from the owners for the participation of their animals in this study.

## Author contributions

ES: Conceptualization, Data curation, Investigation, Methodology, Project administration, Writing – original draft, Writing – review & editing. GP: Data curation, Investigation, Writing – review & editing. VP: Data curation, Investigation, Project administration, Writing – review & editing. IS: Investigation, Methodology, Project administration, Writing – review & editing. ZP: Investigation, Project administration, Writing – review & editing. GK: Conceptualization, Investigation, Project administration, Supervision, Writing – review & editing.

## Glossary

AEDs: Antiepileptic Drugs
ISO: Isoflurane
RSE: Refractory status epilepticus
SRSE: Super-refractory status epilepticus
ICU: Intensive care unit
R: Responder
NR: Non-responder
StE: Structural epilepsy
CE: Cryptogenic epilepsy
RE: Reactive epilepsy
IE: Idiopathic epilepsy
PaCO_2_: Partial pressure of carbon dioxide
EtCO_2_: End-dial carbon dioxide
SE: Status epilepticus
PB: Phenobarbital
LEV: Levetiracetam
KBr: Potassium bromide
IMP: Imepitoin
MDZ: Midazolam
DZP: Diazepam
DEX: Dexmedetomidine
KET: Ketamine
PHT: Phenytoin
No: Number
GABA A: γ-Aminobutyric acid type A
MUO: Meningoencephalitis of unknown origin
NA: Not available
